# Identifying *Mazama gouazoubira* (Artiodactyla; Cervidae) chromosomes involved in rearrangements induced by doxorubicin

**DOI:** 10.1590/1678-4685-GMB-2016-0275

**Published:** 2017-06-05

**Authors:** Iara Maluf Tomazella, Vanessa Veltrini Abril, José Maurício Barbanti Duarte

**Affiliations:** 1 Universidade Estadual Paulista (Unesp), Faculdade de Ciências Agrárias e Veterinárias, Câmpus Jaboticabal, Departamento de Zootecnia, Núcleo de Pesquisa e Conservação de Cervídeos (NUPECCE), Jaboticabal, SP, Brazil; 2 Universidade Federal de Mato Grosso (UFMT), Campus Universitário do Araguaia, Instituto de Ciências Biológicas e da Saúde (ICBS), Pontal do Araguaia, MT, Brazil

**Keywords:** Chromosomal aberrations, chromosome evolution, brown brocket deer, G-banding

## Abstract

The process of karyotype evolution in Cervidae from a common ancestor (2n = 70, FN = 70) has been marked by complex chromosomal rearrangements. This ancestral karyotype has been retained by the current species *Mazama gouazoubira* (Fischer 1814), for which a chromosomal polymorphism (Robertsonian translocations and the presence of B chromosomes) has been described, presumably caused by a chromosome fragility. Thus, this study has identified doxorubicin-induced chromosome aberrations and mapped the regions involved in breaks, which may be related to the chromosome evolution process. G-banding pattern showed that 21 pairs of chromosomes presented chromosomal aberrations, 60% of the total chromosome number of the species *M. gouazoubira*. Among chromosomes that carry aberrations, the region where they were most frequently concentrated was distal relative to the centromere. These data suggest that certain chromosomal regions may be more susceptible to chromosome fragility and consequently could be involved in karyotype differentiation in species of the family Cervidae.

## Introduction

The genus *Mazama* (Rafinesque 1817) belongs to family Cervidae and order Artiodactyla, and consists of ten species with broad morphological and cytogenetic diversity, especially the latter (Duarte and Merino, 1997; Duarte and Jorge, 2003; Abril *et al.*, 2010; Merino and Rossi, 2010). Deer are used as a model for studies on chromosome evolution and speciation, in particular, two genera: *Muntiacus* (Rafinesque 1815) with 2n ranging from 6-7 to 46; and *Mazama* between 32 and 70 chromosomes (Neitzel, 1987; Yang *et al.*, 1997; Abril *et al.*, 2010). The extensive chromosomal diversification presented by species of *Mazama* can be understood by analysing the karyotype evolution that occurred in this genus, which underwent complex interspecific rearrangements (Fontana and Rubini, 1990). This karyotypic variation can be explained by the hypothesis of chromosome fragility proposed by Duarte and Jorge (1996) and corroborated by Vargas-Munar *et al.* (2010), who observed that the chromosomes of the genus *Mazama* are more unstable than chromosomes of the species *Blastocerus dichotomus* (Illiger 1815). Moreover, it has been observed that *Mazama gouazoubira* (Fischer 1814) has the highest rates of chromosomal aberrations induced by doxorubicin among the three species of the genus *Mazama* that have been analysed: *Mazama americana* (Erxleben 1777), *M. gouazoubira* and *Mazama nana* (Hensel 1872).

The brown brocket deer *M. gouazoubira* is a small to medium-sized deer and its coat varies in colour from brown to grey (Black-Décima *et al.*, 2010). In Brazil, the brown brocket deer can be found in all regions except the Amazon region, showing great ecological plasticity, which is related to the occurrence of important morphological and genetic modifications (Pinder and Leeuwenberg, 1997; Rodrigues *et al.*, 2014). The species is characterised by its retention of the ancestral karyotype of Cervidae (2n = 70, FN = 70) with 68 acrocentric autosomal chromosomes, an acrocentric X chromosome and a metacentric Y chromosome (Neitzel, 1987; Fontana and Rubini, 1990). In this species, chromosomal polymorphism is often observed, caused by the presence of B chromosomes and the occurrence of Robertsonian translocations, which could be related to chromosome fragility in this species (Vargas-Munar *et al.*, 2010). Chromosome fragility facilitates the occurrence of chromosome breaks and exchanges, which can induce the formation of new species following geographic isolation over a short period of time (Glover and Stein, 1988).

Doxorubicin, also known as Adriamycin, is a substance belonging to the class of drugs called anthracyclines, which are used in chemotherapy and can be used as a mutagenic agent in studies on mutagenicity (Tan *et al.*, 1973; Blum and Carter, 1979; Young *et al.*, 1981; Resende *et al.*, 2010). In addition to producing highly toxic free radicals, doxorubicin cause random breaks in DNA through the inhibition of topoisomerase II and helicase activity, interfering in the separation of double-stranded DNA (Kitada *et al.*, 1994; Coquelle *et al.*, 1997; Quiles *et al.*, 2002; Islaih *et al.*, 2005). Reactive free radicals are considered to be mediators of the toxicity induced by doxorubicin, which is characterised by changes in morphology and mitochondrial function (Bachmann *et al.*, 1975; Porta *et al.*, 1983). The oxidative process caused by free radicals induces DNA damage and can be repaired by the system of cell repair; however, if repair does not occur, oxidative damage results in certain pathophysiological processes, such as mutagenesis, carcinogenesis and cell aging (Dizdaroglu *et al.*, 2008; Halliwell and Gutteridge, 2015). When the damage caused by doxorubicin to DNA is not repaired it accumulates, resulting in mutational events and chromosomal aberrations (CAs), in both healthy and tumour cells (Islaih *et al.*, 2005). It has been suggested that the mutagenicity of doxorubicin could be the result of damage caused by catalysing NADH dehydrogenase in close proximity to DNA (Akman *et al.*, 1992).

Since the species *M. gouazoubira* retains the ancestral karyotype (Neitzel, 1987; Fontana and Rubini, 1990), is the basis for the formation of karyotypes of other deer species and has shown high rates of CAs when its cells are submitted to doxorubicin (Vargas-Munar *et al.*, 2010), it was chosen as the object of this study. Identifying chromosomes with high rates of CAs could indicate which chromosomes are involved in the chromosomal changes that have occurred during the karyotype evolution of deer. Thus, this study aimed to identify chromosomes with chromosomal aberrations and map regions of breaks induced by doxorubicin in chromosomes of *M. gouazoubira* by analysing the frequency and the different types of breaks that occurred in each chromosome.

## Materials and Methods

Blood samples were collected from six deer (three females: F1, F2 and F3; three males: M1, M2 and M3) maintained in captivity by the Deer Research and Conservation Centre (NUPECCE) of the Department of Animal Science, School of Agricultural and Veterinary Sciences (FCAV), São Paulo State University (UNESP), Jaboticabal, SP, Brazil. Lymphocyte cultures were established using these blood samples (Moorhead *et al.*, 1960): control cell cultures (CC), and doxorubicin-treated cell cultures (TC). To induce CAs, doxorubicin hydrochloride (Adriblastina® Pharmacia) at a concentration of 0.25 μg/mL was added to the final 24 h of culture (Antunes and Takahashi, 1998). The CC and TC of each deer sample were performed in triplicate.

Data analysis was performed in three stages: 1) calculation of the mitotic index (MI): this was determined by the ratio between the number of metaphases identified in 2,000 cells per culture, analysing 6,000 cells per deer (Galloway *et al.*, 1994; Takahashi, 2003); 2) analysis of chromosomal aberrations by conventional Giemsa staining: the number of CAs (NCAs) and the number of metaphases shown by the CAs (NMCAs) were assessed. In both analyses, the values obtained for the CC were compared with the values obtained for the TC. Next, the CAs were identified and quantified as: ring, dicentric chromosome, chromatid gap, chromosome gap, chromatid break, chromosome break, triradial form, quadriradial form and rearrangement (Evans and O'Riordan, 1975; Savage, 1975; Savage and Simpson, 1994); 3) identification and mapping of chromosomal regions by G-banding and calculation of the percentage of chromosomes with CAs: for each deer sample, the metaphases of the TC submitted to the G-banding technique were analysed (Seabright, 1971, with modifications), and in each metaphase, the number of chromosomes and which pairs carried CAs was ascertained, in order to determine the NCAs and NMCAs. The chromosomal biometrics of all the deer were used to calculate the mean percentage of chromosomes that carried CAs. After identifying chromosomes with CAs, the regions where breaks occurred were mapped on each of these chromosomes. To achieve this, the chromosomes were divided into three regions in relation to the centromere: proximal, medial and distal. In this step, the idiogram proposed by Neitzel (1987) was used for comparison. Then, a nonparametric Chi square test (χ2) was performed at a significance level of 0.05 (α = 5%) to determine whether the distribution of CAs observed on the chromosomes was random or organised. In steps one and two, the mean values of the three replicates were evaluated statistically by analysis of variance for completely randomised experiments, for each deer sample, using the Tukey test (p < 0.05). The statistical analyses were performed in GRAPHPAD PRISM 7.0 software.

## Results

### Mitotic index

No statistically significant differences (p > 0.05) in values were observed for the mitotic index (MI) between the doxorubicin-treated cell cultures (TC) and control cell cultures (CC). This indicates the absence of cytotoxicity under the experimental conditions of the cultures using different deer samples. No significant difference (p > 0.05) was observed between the mean MI values of all male and female cell cultures, indicating that the sex of the deer did not influence the MI.

### Chromosome aberrations by conventional Giemsa staining

**Figure 1:**
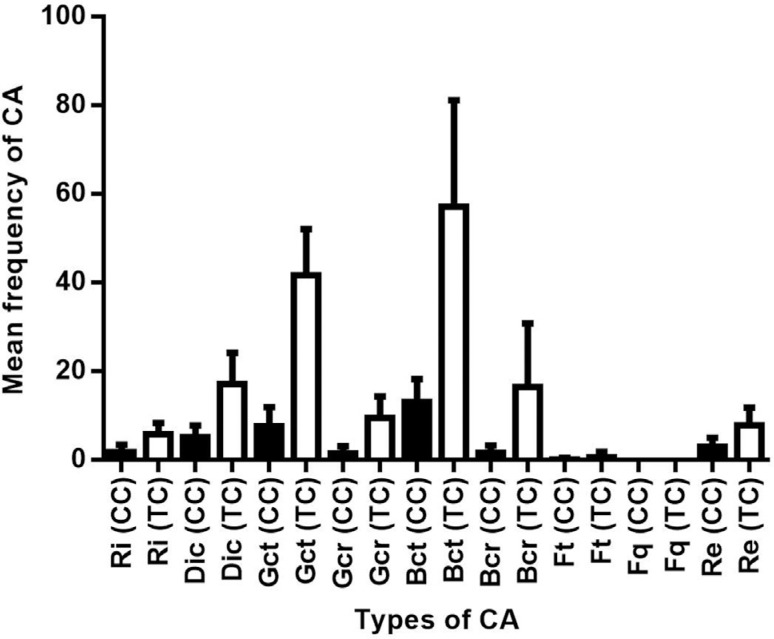
Mean frequency of the types of chromosomal aberrations (CAs) analysed in control cell cultures (CC, black bars) and doxorubicin-treated cell cultures (TC, white bars) for female and male deer, in each experiment. Types of chromosomal aberrations: ring (Ri), dicentric (Dic), chromatid gap (Gct), chromosome gap (Gcr), chromatid break (Bct), chromosome break (Bcr), triradial form (Ft), quadriradial form (Fq) and rearrangement (Re).

**Figure 2:**
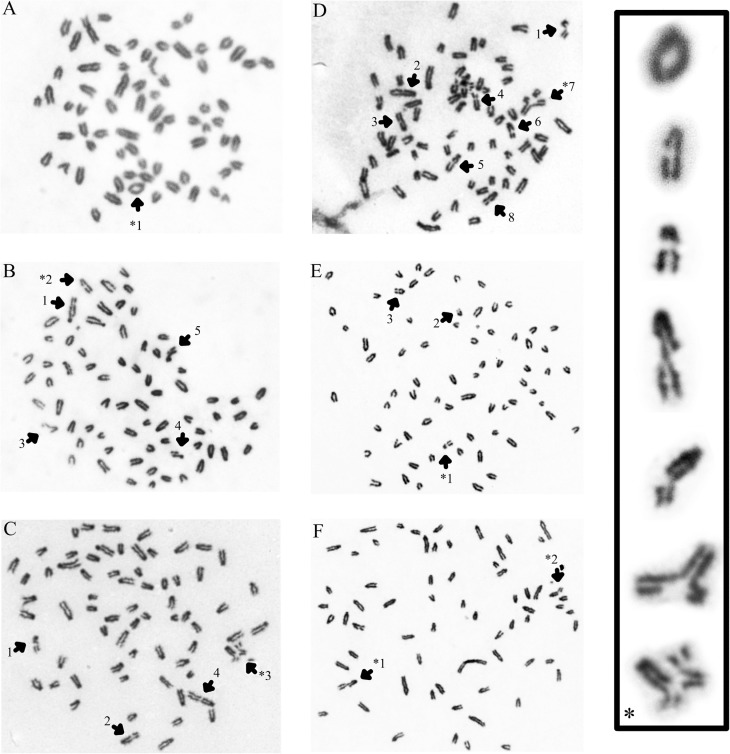
Different types of chromosomal aberrations obtained in doxorubicin-treated cell cultures. (A) 1 = ring; (B) 1 and 2 = chromatid gap, 3, 4 and 5 = chromatid break; (C) 1 = chromatid break, 2 = chromatid gap, 3 = rearrangement and 4 = chromosome gap; (D) 1 = chromosome break, 2, 3, 5, 6 and 8 = chromatid gap, 4 = chromosome gap and 7 = triradial form; (E) 1, 2 and 3 = chromosome gap; (F) 1 = chromatid break and 2 = chromosome break. Chromosome preparations were stained with Giemsa and visualised at 1000x magnification. (*) Details of some chromosomal aberrations are shown at higher magnification.

Regarding the NCAs and NMCAs, a statistically significant difference (p < 0.05) was observed between the CC and TC, such that TC showed a significant increase in NCAs and NMCAs for both male and female deer samples. No significant differences (p > 0.05) were observed in the NCAs and NMCAs between male and female samples in the CC or the TC, indicating sex had no influence on CAs or MCAs (Table 1). Regarding the CC, the most frequent types of CAs observed were chromatid break and chromatid gap followed by dicentric chromosome, rearrangement, ring, chromosome break, chromosome gap and triradial form, on both male and female deer chromosomes. In the TC, the most frequent types of CAs were chromatid break and chromatid gap, followed by dicentric chromosome, chromosome break, chromosome gap, rearrangement, ring and triradial form, on both male and female deer chromosomes. Quadriradial forms were not detected in any of the lymphocyte cultures analysed (Figure 1). Figure 2 presents some of the CAs detected in metaphases of the TC. using Giemsa stain.

**Table 1:** Percentage of the number of chromosomal aberrations (NCAs) and the number of metaphases carrying chromosomal aberrations (NMCAs) in control cell cultures (CC) and doxorubicin-treated cell cultures (TC). Female (F1, F2 and F3), male (M1, M2 and M3).

Deer	NCAs (%)		NMCAs (%)	
	CC	TC	CC	TC
F1	15.57	20.57	17.20	19.14
F2	15.57	20.04	15.92	19.51
F3	16.98	15.68	17.20	15.38
M1	17.45	15.99	16.56	16.51
M2	17.92	14.18	17.83	15.20
M3	16.51	13.54	15.29	14.26

100 metaphases per cell culture were analysed totalling 300 metaphases per deer.

### Identification and mapping of chromosomal regions by G-banding and calculation of the percentage of chromosomes with CAs

**Figure 3:**
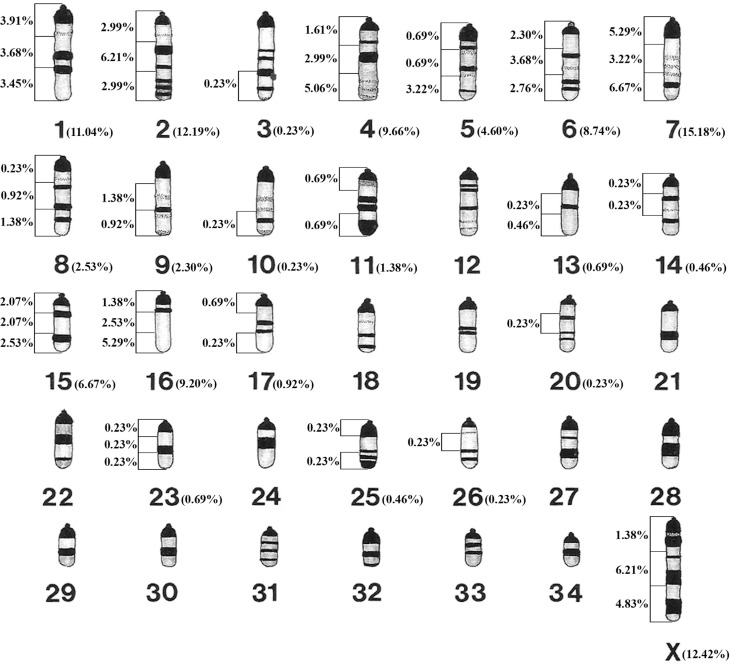
Idiogram of the G-banding pattern of *Mazama gouazoubira* chromosomes, indicating the location and frequency of chromosomal aberrations induced by doxorubicin in each pair and each chromosomal region. Idiogram proposed by Neitzel (1987), with modifications.

CAs were not detected on chromosome pairs 12, 18, 19, 21, 22, 24, 27, 28, 29, 30, 31, 32, 33, 34 or the Y sex chromosome for all the samples analysed. Among all the deer samples analysed, chromosome pair 7 showed the highest frequency of CAs (15.18%), followed by the pairs X (12.42%), 2 (12.19%), 1 (11.04%), 4 (9.66%), 16 (9.20%), 6 (8.74%), 15 (6.67%), 5 (4.60%), 8 (2.53%), 9 (2.30%), 11 (1.38%), 17 (0.92%), 13 and 23 (0.69%), 14 and 25 (0.46%), 3, 10, 20 and 26 (0.23%) (Figure 3). Mapping of the regions where the breaks occurred showed that the distal chromosomal region (relative to the centromere) presented the highest frequency of CAs induced by doxorubicin compared with the proximal and medial regions.

The Chi square test (Table 2) showed that chromosome pairs 1, 2, 4, 6, 7, 15, 16 and the X sex chromosomes carried higher than statistically expected CAs, indicating that the CAs on these chromosomes did not occur at random. This finding suggests the presence of chromosomal instability resulting from the chromosome fragility previously described in this species. In contrast, the CAs detected on chromosome pairs 3, 5, 8, 9, 10, 11, 13, 14, 17, 20, 23, 25 and 26 showed no statistically significant differences (p > 0.05), indicating that the quantity of CAs on these chromosomes is within expected values.

**Table 2:** Values of the probability of each chromosome pair carrying chromosomal aberrations.

Chromosome pairs	Expected value (%)	Observed value (%)	calculated χ^2^
1	4.28	11.04	10.67
2	3.94	12.19	17.27
3	3.74	0.23	3.29
4	3.60	9.66	10.20
5	3.50	4.60	0.34
6	3.42	8.74	8.27
7	3.34	15.18	41.97
8	3.28	2.53	0.17
9	3.24	2.30	0.27
10	3.16	0.23	2.71
11	3.08	1.38	0.93
12	3.01	0	3.01
13	2.97	0.69	1.75
14	2.93	0.46	2.08
15	2.87	6.67	5.03
16	2.83	9.20	14.33
17	2.79	0.92	1.25
18	2.71	0	2.71
19	2.66	0	2.66
20	2.60	0.23	2.16
21	2.56	0	2.56
22	2.52	0	2.52
23	2.47	0.69	1.28
24	2.44	0	2.44
25	2.40	0.46	1.56
26	2.35	0.23	1.91
27	2.30	0	2.30
28	2.23	0	2.23
29	2.19	0	2.19
30	2.12	0	2.12
31	2.02	0	2.02
32	1.97	0	1.97
33	1.89	0	1.89
34	1.64	0	1.64
X	4.95	12.42	11.27

Tabulated Chi square (χ^2^) = 3.84; significance level of 0.05 (α = 5%).

Twenty-one pairs of chromosomes carried CAs; 60% of the chromosome number of the species *M. gouazoubira*. Analysis of the data showed that the CAs were most frequently concentrated in certain regions of specific chromosomes, indicating that the CAs are not randomly distributed.

## Discussion

Since the species *M. gouazoubira* has retained the ancestral karyotype of Cervidae, the basis for the origin of karyotypes of other species, this study used this species to identify the chromosomes and chromosomal regions carrying CAs induced by doxorubicin, and analysed the frequency and different types of CAs observed on its chromosomes. The identification of chromosomes that carry CAs is an advance in the study of chromosome evolution in cervids, enabling researchers to correlate these chromosomes with karyotypic rearrangements that have occurred during the process of speciation in the family Cervidae.

Doxorubicin, the mutagenic agent used to induce the CAs in this study, works through different mechanisms, all of which cause cell damage. Chromosome damage caused by this mutagen occurs during certain phases of the cell cycle and is the result of doxorubicin binding strongly to DNA during the replication process. This strong binding is due to the intercalation of doxorubicin between pairs of DNA bases, forming a stable complex which prevents the fixation and, consequently, the activity of DNA polymerase (DiMarco *et al.*, 1964; Gale *et al.*, 1973). The different types of CAs analysed in this study were probably induced by the formation of such stable complexes. Another mechanism of action of doxorubicin is its production of highly reactive free radicals, which cause changes in the nitrogenous bases of DNA (Lown, 1983; Akman *et al.*, 1992). This oxidative damage results in a wide variety of biological effects, including cell death, mutagenesis and gene amplification. It is known that gene amplification caused by mutagens occurs in fragile sites, causing chromosomal instability (chromosome fragility). Mutagenic agents causing oxidative stress, such as doxorubicin, can induce DNA breaks and chromosomal translocations in the sequences of fragile sites (Hunt *et al.*, 1998). According to Hunt *et al.* (1998), doxorubicin seems to cause damage to fragile sites, a fact that contradicts studies showing that it causes CAs in randomly distributed regions on chromosomes (Newsome and Littlefield, 1975; Coquelle *et al.*, 1997). The data presented by Hunt *et al.* (1998) corroborate the results obtained in this study, which showed that doxorubicin did not cause random CAs on *M. gouazoubira* chromosomes. Rather, CAs were more frequently mapped at specific sites. In this study, certain chromosomes were seen to carry more CAs than others, at frequencies higher than those expected for random distribution. Moreover, on chromosomes that carried CAs, these occurred more frequently in certain regions, a fact that could be related to chromosome fragility in the species concerned.

Regarding the types of CAs detected in the TC, for both female and male deer, the most frequently observed types were chromatid break and chromatid gap. Similar results have also been observed in studies on other mammalian groups, which reported higher incidences of chromatid-type CAs (Newsome and Littlefield, 1975; Antunes and Takahashi, 1998; Gülkaç *et al.*, 2004). High frequencies of chromatid damage are justified by the fact that the lymphocytes were exposed to a mutagenic agent during the S or G_2_ phases, when the chromosomes have two sister chromatids (Evans and O'Riordan, 1975).

It is known that the X sex chromosome of some mammalian species presents an instability resulting from chromosome fragility (Slota *et al.*, 2000). Similar results were obtained by Llambí *et al.* (2008), who observed that the X chromosome is highly fragile in bovines, unlike the Y chromosome. Besides detecting CAs on the X sex chromosome in individuals of the species *Ovis aries* (Linnaeus 1758), Ahmad *et al.* (2008) showed that X chromosomes of females were generally more fragile than the X chromosomes of males. Similar findings were not supported by the data obtained herein, since no significant differences were verified between CA rates in the X chromosomes of female and male *M. gouazoubira*.

The greater chromosome fragility observed in *M. gouazoubira* when submitted to the action of doxorubicin, compared with other species of the genus, may be related to the fact that the species retains the ancestral karyotype. It seems likely that this karyotype has fragile sites for rearrangements that were lost in more derived karyotypes during chromosome evolution of the family Cervidae (Neitzel, 1987; Fontana and Rubini, 1990; Abril *et al.*, 2010; Vargas-Munar *et al.*, 2010). Among the rearrangements that have occurred during karyotypic change and evolution in the family Cervidae, numerous inversions and centric and tandem fusions can be cited that decreased the diploid number in the process of speciation (Fontana and Rubini, 1990; Abril *et al.*, 2010). It is possible that fragile sites contained in the ancestral karyotype have been preserved in the current karyotype of the brown brocket deer and its genome is still subject to chromosome fragility. Indications of this are evident in the high rate of intraspecific polymorphism resulting from centric fusions involving different chromosomes (Duarte and Jorge, 1996; Valeri, 2014). It is reasonable to assume these centric fusions are composed of chromosomes that have sequences of fragile sites, which make them more susceptible to the occurrence of this rearrangement. Four chromosome pairs (X, 4, 7 and 16), characterised as carrying CAs in this study, were identified as components of centric fusions studied by Valeri (2014) in a population from the Brazilian Pantanal. These fusions favour the accumulation of constitutive heterochromatin present in the pericentromeric region. Neitzel (1987) suggested that pericentromeric heterochromatin is related to the structural organisation of chromosomes of species of the family Cervidae. Some studies show that CAs, breakpoints and fragile sites have been detected in the intercalary heterochromatin, which is a type of euchromatin that possesses the properties of constitutive heterochromatin (Marlhens *et al.*, 1986; Laird *et al.*, 1987; Miró *et al.*, 1987). In contrast to these findings, and despite the frequent observation of centric fusions in *M. gouazoubira*, this study showed that the majority of the CAs did not map in areas of pericentromeric constitutive heterochromatin.

Pevzner and Tesler (2003) proposed a model to explain the chromosome evolution of mammals, known as “fragile breakage”, which stated that in the mammalian genome, there are fragile regions (short sequences) and solid regions (long sequences) that present different probabilities of breakage. The solid regions are protected against the occurrence of rearrangements, since these blocks correspond to functional genes, which play vital roles in the development of an organism. In contrast, the fragile regions are prone to break and reorganise themselves, representing, for example, a small proportion of blocks of genes involved in adaptive capacity due to the high evolutionary cost of eliminating mutations that affect multiple genes simultaneously (Peng *et al.*, 2006; Becker and Lenhard, 2007). Thus, it is likely that the CAs identified in this study are located in fragile regions of *M. gouazoubira* chromosomes. This fragility leads to a chromosomal instability, making some regions more susceptible to the occurrence of chromosomal rearrangements, which could be involved in the impressive karyotype evolution of species of deer, especially among species of the genus *Mazama*.

The identification of chromosomes with higher rates of aberrations in this work is the starting point for future studies on the process of karyotype evolution in the genus *Mazama*. Using chromosomal probes of these known pairs should lead to precise knowledge on how they behave during karyotype differentiation between species. It should then be possible to determine whether the high rate of breaks in these pairs caused by doxorubicin, a random mutagenic agent, is related to the rearrangements that have occurred during chromosome evolution.
